# The Dynamics of Liver Function Test Abnormalities after Malaria Infection: A Retrospective Observational Study

**DOI:** 10.4269/ajtmh.17-0754

**Published:** 2018-02-12

**Authors:** John Woodford, G. Dennis Shanks, Paul Griffin, Stephan Chalon, James S. McCarthy

**Affiliations:** 1 QIMR Berghofer Medical Research Institute, Brisbane, Australia;; 2 Royal Brisbane and Women’s Hospital, Brisbane, Australia;; 3 The University of Queensland, Brisbane, Australia;; 4 Australian Army Malaria Institute, Brisbane, Australia;; 5 Department of Medicine and Infectious Diseases, Mater Hospital and Mater Medical Research Institute, Brisbane, Australia;; 6 Medicines for Malaria Venture, Geneva, Switzerland

## Abstract

Liver dysfunction has long been recognized as a clinical feature of malaria. We have observed delayed elevation in the transaminase portion of liver function tests (LFTs) after treatment in some participants undergoing induced blood stage malaria infection. We sought to determine whether similar LFT elevations occur after naturally acquired infection. We performed a retrospective audit of confirmed cases of *Plasmodium falciparum* and *Plasmodium vivax* in Queensland, Australia, from 2006 to 2016. All LFT results from malaria diagnosis until 28 days after diagnosis were collected with demographic and clinical information to describe longitudinal changes. The timing of peak LFT elevations was classified as early (0–3 days), delayed (4–11 days), or late (12–28 days) with respect to the day of diagnosis. Among 861 cases with LFT evaluated, an elevated bilirubin level was identified in 12.4% (*N* = 107/861), whereas elevated alanine transaminase (ALT) and aspartate transaminase levels were observed in 15.1% (*N* = 130/861) and 14.8% (*N* = 127/861) of cases, respectively. All peak bilirubin results occurred in the early period, whereas ALT elevations were biphasic, with elevations in the early and delayed periods, with 35.4% (*N* = 46/130) of cases delayed. Univariate and paired stepwise logistic regression analyses were performed to investigate factors associated with the incidence and timing of transaminase elevation. A raised ALT level at diagnosis was strongly associated with the timing of transaminase elevation. No other demographic, parasitic, or treatment factors were associated. Liver function test abnormalities are likely an inherent although variable aspect of human malaria, and individual-specific factors may confer susceptibility to hepatocyte injury.

## INTRODUCTION

Abnormalities in liver function have been described but relatively little investigated compared with other aspects of clinical malaria. Reporting of malaria-associated liver injury in the literature is heterogeneous and primarily focused on biochemical measurement of liver function tests (LFTs), such as bilirubin and the transaminases alanine transaminase (ALT) and aspartate transaminase (AST). There are few thorough longitudinal descriptions of malaria-associated liver injury.

Surveys of falciparum malaria have reported lower rates of jaundice at presentation in endemic populations (2.6–5.3% of cases) compared with epidemic malaria (11.5–62% of cases).^1–7^ Other *Plasmodium* species have been associated with a relatively lower rate of jaundice compared with falciparum.^8,9^ Mild elevations of transaminases are common, although more significant elevations associated with multiorgan dysfunction have also been reported.^10–14^ The syndrome of “malarial hepatopathy” has been recently proposed, being defined as a bilirubin level > 2.5 times upper limit of normal (ULN) with associated transaminase elevation > 3 × ULN (where ALT is considered the more liver-specific enzyme).^1,2,15–19^ Although the clinical significance of malarial hepatopathy has not been fully elucidated, it represents an attempt to further describe malaria-associated liver injury and has been associated with more severe disease and other organ dysfunction.^1^ The relationship of malarial hepatopathy to antimalarial treatment needs to be evaluated to differentiate it from clinically significant drug-induced liver injury meeting Hy’s Law, which has a similar definition.^20^ Malarial hepatopathy has been reported in 2.6–45% of all malaria cases and up to 87.5% of cases presenting with clinical jaundice.^1,2,4,13,18,21,22^ Kupffer cell hyperplasia, hemozoin loading, and monocyte infiltration are the most frequently reported histological findings in malaria-associated liver injury; all seem to resolve after antimalarial treatment.^2,4,12,23–27^

Although the incidence of abnormal LFTs has been described in multiple studies, the temporal pattern of LFT abnormalities has been less explored. Available data indicate that LFT abnormalities due to malaria typically resolve after 1–2 weeks compared with 6–8 weeks after acute hepatitis A, B, or C.^9,16^

Peaks in transaminase levels after treatment have also been recently reported; these could represent a distinct type of malaria-associated liver injury.^28–31^ In a small retrospective comparative European study, asymptomatic transaminase elevation was observed in returned travelers hospitalized for malaria after treatment with the artemisinin-based combination therapy artemether–lumefantrine. The authors postulated that the rapid parasiticidal effect of the antimalarial treatment may promote accelerated hepatic heme loading and oxidative stress.^28^ Liver injury through this proposed mechanism is not exactly an adverse drug reaction but rather may be influenced by factors, such as parasite burden, rate of clearance, and host heme metabolism. In prospective trials using the induced blood stage malaria (IBSM) model in healthy malaria-naive participants, asymptomatic moderate (2.5–5.0 × ULN) to more rarely severe (> 5.1 × ULN) elevations of transaminase enzymes have been reported in a subset of participants. These isolated transaminase elevations are associated with an ALT/AST ratio > 1 and a normal bilirubin level, peaking 4–12 days after treatment, and have been reported following multiple antimalarial compounds in both falciparum and vivax malaria models.^29,30^ Similar elevations have been observed after sporozoite malaria challenge.^31,32^ Delayed transaminase elevation may be attributed to the antimalarial compound, the use of acetaminophen for symptom relief,^33^ the malaria parasite, or host factors associated with parasite clearance. Although the relative contribution of each of these factors is uncertain, it is important to understand their relative contribution, given the implications that unexplained transaminase elevation may have on antimalarial drug development.

To further characterize the spectrum of malaria-associated liver stress/injury, we performed a retrospective audit of cases of *Plasmodium falciparum* and *Plasmodium vivax* infection in Queensland, Australia. In this report, we describe the incidence, degree, and timing of abnormal LFTs in a population of patients with naturally acquired malaria infection. Cases with LFT outcomes of interest were specifically investigated to describe the temporal relationship between antimalarial treatment and transaminase elevation. We also analyzed factors that may contribute to transaminase elevation, specifically the timing of peak elevation after treatment.

## METHODS

### Subjects.

We included patients of any age with microscopy-confirmed cases of *P. falciparum* and *P. vivax* malaria on the Pathology Queensland electronic database (AUSLAB Clinical and Scientific Information System) between January 1, 2006 and June 1, 2016, an interval that covers the period of electronic pathology record keeping in the State. Inpatient and outpatient data were included. Duplicate results, defined as any malaria positive test in the same individual for the same *Plasmodium* species collected within 7 days, were excluded. The reference case for inclusion was considered the earliest test collected at a Queensland Health facility. *Plasmodium* species coinfections were included. *Plasmodium falciparum* was considered the reference infection unless *P. vivax* monoinfection was diagnosed within 7 days earlier.

### Data collection.

Data on demographics (age at diagnosis, gender, and date and location of pathology sample collection), parasitemia (by microscopy at the time of diagnosis), and LFTs (ALT, AST, and total bilirubin) from the day of malaria diagnosis (Day 0) to 28 days after diagnosis were collected from the Pathology Queensland database for all cases. Where multiple results were found for a single day, the earliest result was recorded. All LFT results were converted to multiples of the ULN to allow for comparison between age and gender groups (Supplemental Table 1).

### Classification of LFTs.

Liver function tests were classified using a modified World Health Organization (WHO) toxicity grade system (Grades 0 to 4), which is used to assess adverse events in clinical trials (Table 1).^34^ Mild (Grade 1) elevations of LFTs were not considered clinically significant in the context of documented malaria (1.25–3 × ULN for ALT and AST, 1.25–2.5 × ULN for bilirubin). Malarial hepatopathy was defined as bilirubin > 2.5 × ULN with a concurrent ALT > 3 × ULN.^1^ The peak LFT measurement and day after malaria diagnosis in which it was measured were used to determine the severity and temporal pattern, respectively. We grouped LFT results into three time periods: early (Day 0 to Day 3), delayed (Day 4 to Day 11), and late (Day 12 to Day 28). Time periods were based on the timing of delayed transaminase elevation observed in healthy nonimmune participants in IBSM trials.^29,30^

**Table 1:** Modified World Health Organization toxicity grade system*

	Mild grade 1†	Moderate grade 2	Severe grade 3	Very severe grade 4
ALT × ULN	1.25–3.0	3.1–5.0	5.1–10.0	> 10.0
AST × ULN	1.25–3.0	3.1–5.0	5.1–10.0	> 10.0
Bilirubin × ULN	1.25–2.5	2.6–5.0	5.1–10.0	> 10.0

ALT = alanine transaminase; AST = aspartate transaminase; ULN = upper limit of normal.

* Cutoff values are adapted from the World Health Organization toxicity grade system.^34^

† Mild (Grade 1) liver function test elevations were not considered clinically significant in the context of documented malaria.

Early, delayed, and late transaminase elevations were defined as having a moderate, severe, or very severe grade peak ALT result within the appropriate time period. The reference normal group was defined as cases with serial ALT measurements < 2 × ULN across contiguous time periods. This cutoff was chosen to increase the size of this group for comparison. An electronic chart audit and a review of Public Health records were conducted to identify the geographical region where malaria was acquired and the type and timing of treatment regimen, where this information was documented.

Treatment regimen was classified as artemisinin-containing or nonartemisinin-containing. The timing of treatment initiation with respect to the day of diagnosis was recorded to determine the treatment delay. Other infections potentially contributing to abnormal LFTs were identified in Pathology Queensland records. This was defined as any positive acute phase testing for dengue, other arboviruses, herpes viruses, viral hepatitis, and other infections (leptospirosis, Q-fever, and Rickettsial disease) occurring within 28 days after malaria diagnosis. Log-transformed ULN data for total bilirubin, ALT, AST and the ratio of ALT/AST at diagnosis, demographics, region of acquisition, log-transformed parasitemia at diagnosis, *Plasmodium* species, presence of other infections, and treatment regimen were compared between groups to identify possible contributing factors to transaminase elevation. Data on the use of potentially hepatotoxic medications were not available.

### Statistical analysis.

Descriptive statistics are presented as mean with standard deviation (SD), median with interquartile range (IQR), or percentage. Cases were grouped by outcomes: early, delayed, and late transaminase elevations or serial normal transaminase results. Parasitemia and LFT × ULN results were log transformed to normalize data distribution. Assessment of normality of distribution was undertaken using the D’Agostino test. All groups were compared using the chi-squared test, Kruskal–Wallis test, or one-way analysis of variance to compare available clinical/demographic data between groups. A *P* < 0.05 was considered statistically significant. A paired stepwise logistic regression model was used in an exploratory multivariate analysis of all clinical/demographic factors available to determine, which were independently predictive of transaminase outcome. Likelihood ratios (LRs) were calculated for variables considered predictive of outcome by the model (*P* < 0.05). No adjustments were made. GraphPad Prism 7.0 (San Diego, CA) was used for descriptive statistics and univariate analysis. JMP Pro 13.2 (Marlow, Buckinghamshire, United Kingdom) was used for the paired stepwise logistic regression analysis.

### Bias.

It is not possible to identify the true frequency of malaria-associated LFT elevations from this retrospective cohort, as many patients with malaria infection did not undergo LFT measurement. The exact timing of peak LFT elevation is not known for many cases because daily testing was not performed. Cases with early LFT abnormalities or other complications may have undergone more frequent monitoring and be overrepresented in the study population.

### Ethics approval.

This study was approved by the Royal Brisbane and Women’s Hospital Human Research Ethics Committee (EC00172; AU/10/4888215).

## RESULTS

### Subject characteristics.

**Figure 1.**
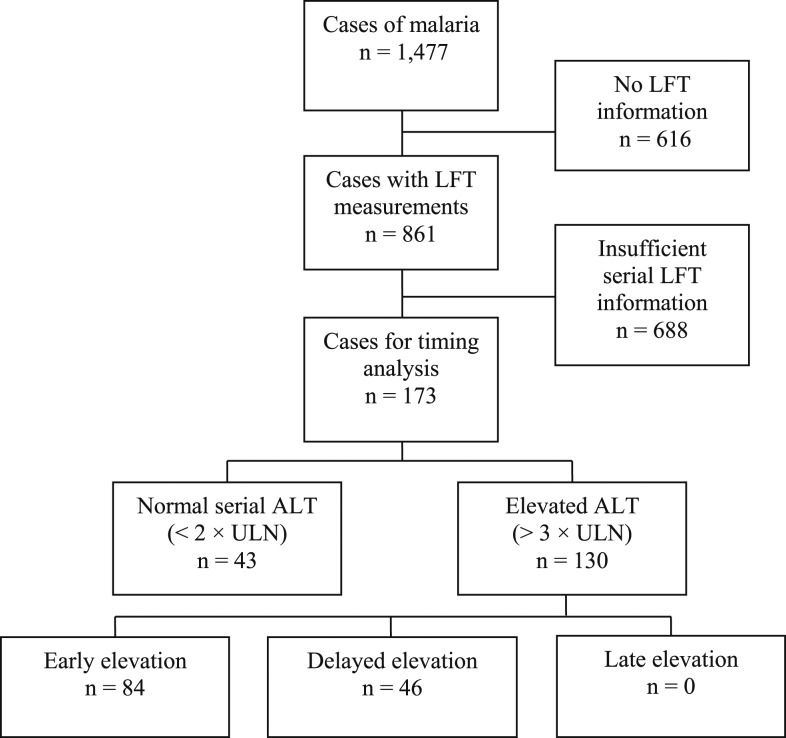
Study population. Confirmed cases of malaria from reference laboratories on the Pathology Queensland database. Time period with respect to date of diagnosis: early (Day 0 to Day 3), delayed (Day 4 to Day 11), and late (Day 12 to Day 28). The ALT level < 2 was considered normal. The ALT level > 3 was considered elevated. ALT = alanine transaminase; AST = aspartate transaminase; LFT = liver function test.

A total of 1,477 individual cases were assessed (Figure 1). Of these, 861 cases had at least one LFT measurement in the 28 days after diagnosis. These cases were used to generate the description of LFTs in malaria infection. A total of 2,189 LFT measurements were reported from all cases with LFT measurements. Of these, 1,686, 348, and 155 measurements occurred in the early, delayed, and late periods, respectively. From the 861 cases with LFT measurements, 688 were not elevated but lacked sufficient serial information to exclude delayed elevation. The remaining 173 cases were used for analysis of the timing of LFT elevations.

Compared with patients who did not have LFT measurement, those who had LFTs collected had a significantly higher rate of infection with *P. falciparum* (49.7% versus 42.5%, *P* = 0.007) and an older age at diagnosis (34.9 years, SD = 16.6 versus 32.7 years, SD = 17.7, *P* = 0.021), but no difference in gender distribution or parasitemia at presentation.

### Description of LFTs.

**Figure 2.**
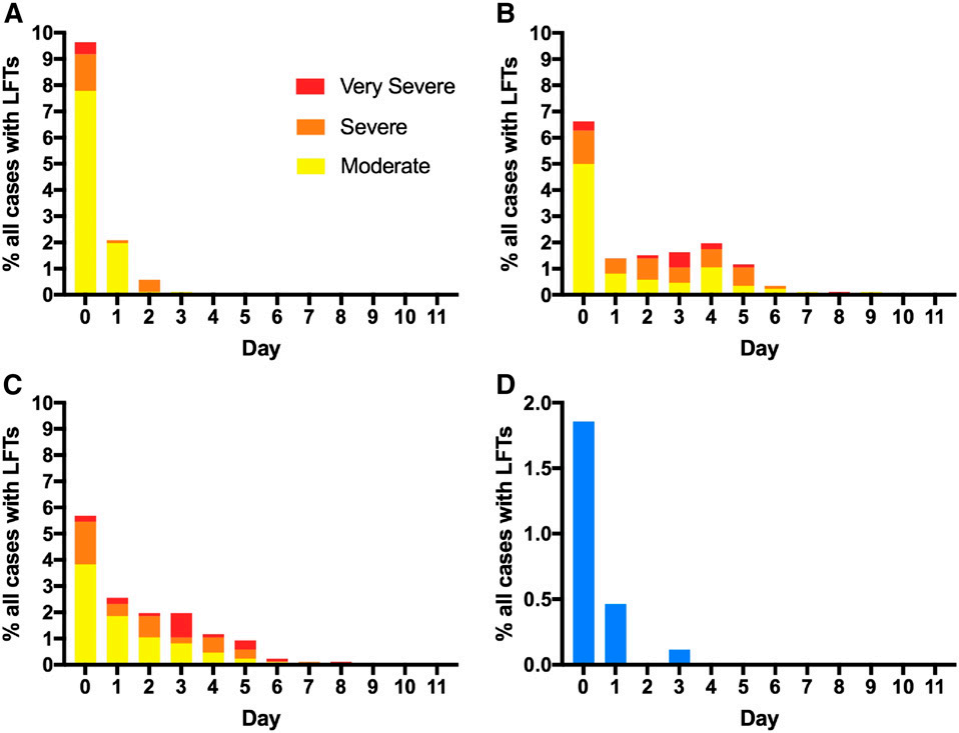
Incidence of abnormal LFTs by day in all cases with LFT measurements (*N* = 861). (**A**) Peak bilirubin (*N* = 107/861). Bilirubin grading: moderate 2.6–5.0 × ULN, severe 5.1–10.0 × ULN, and very severe > 10.0 × ULN. (**B**) Peak ALT (*N* = 130/861). (**C**) Peak AST (*N* = 127/861). ALT and AST grading: moderate 3.1–5.0 × ULN, severe 5.1–10.0 × ULN, and very severe > 10.0 × ULN. (**D**) Malarial hepatopathy (*N* = 21/861, concurrent bilirubin level > 2.5 × ULN and ALT level > 3 × ULN). Day 0 was the day of diagnosis. ALT = alanine transaminase; AST = aspartate transaminase; LFT = liver function test; ULN = upper limit of normal. This figure appears in color at www.ajtmh.org.

The level of bilirubin was elevated > 2.5 × ULN in 12.4% (*N* = 107/861) of all cases with LFT measurements (Table 2). Bilirubin elevation was moderate in most cases, although 21/861 cases were severe or very severe with a maximum value of 20.5 × ULN. All cases with bilirubin elevation peaked before Day 3 (Figure 2). Malarial hepatopathy was found in 2.4% (*N* = 21/861) of all cases.

**Table 2:** Peak LFT results of all cases with LFT measurements classified by the modified WHO toxicity grade system

	WHO grade	All cases with LFTs collected (*N* = 861)	*Plasmodium falciparum* cases with LFTs collected (*N* = 428)	*Plasmodium vivax* cases with LFTs collected (*N* = 433)	Comparison *P. falciparum* and *P. vivax* cases
Peak bilirubin (*n*)	Moderate % (*n*/*N*)	10.0 (86/861)	12.1 (52/428)	7.9 (34/433)	*P* = 0.003
	Severe % (*n*/*N*)	2.0 (17/861)	2.8 (12/428)	1.2 (5/433)	
	Very severe % (*n*/*N*)	0.4 (4/861)	0.9 (4/428)	0 (0/433)	
	Total % (*n*/*N*)	12.4 (107/861)	15.9 (68/428)	9.0 (39/433)	
Peak ALT (*n*)	Moderate % (*n*/*N*)	8.8 (76/861)	10.0 (43/428)	7.6 (33/433)	*P* < 0.001
	Severe % (*n*/*N*)	4.7 (41/861)	6.8 (29/428)	2.8 (12/433)	
	Very severe % (*n*/*N*)	1.5 (13/861)	2.6 (11/428)	0.5 (2/433)	
	Total % (*n*/*N*)	15.1 (130/861)	19.4 (83/428)	10.9 (47/433)	
Peak AST (*n*)	Moderate % (*n*/*N*)	8.4 (72/861)	12.1 (52/428)	4.6 (20/433)	*P* < 0.001
	Severe % (*n*/*N*)	4.2 (36/861)	7.0 (30/428)	1.4 (6/433)	
	Very severe % (*n*/*N*)	2.2 (19/861)	4.0 (17/428)	0.5 (2/433)	
	Total % (*n*/*N*)	14.8 (127/861)	23.1 (99/428)	6.5 (28/433)	

ALT = alanine transaminase; AST = aspartate transaminase; LFT = liver function test; WHO = World Health Organization. Fisher’s exact test comparing the total number of *P. falciparum* and *P. vivax* cases with bilirubin, ALT, or AST abnormalities. “Peak” refers to the highest recorded LFT result of an individual in the 28 days after malaria diagnosis. (*N*) refers to the total sample size, and (*n*) refers to the number of cases with an elevated LFT for each of the parameters measured.

An elevated ALT level > 3 × ULN was identified in 15.1% (*N* = 130/861) of all cases with LFT measurements. New cases were identified up to Day 9 after diagnosis. Moderate, severe, and very severe peak elevations were observed in 76/130, 41/130, and 13/130 cases, respectively (Table 2). The peak ALT elevation identified was 25.8 × ULN and occurred on Day 4. Of the 130 cases with an ALT level > 3 × ULN, 64.6% peaked in the early period (*N* = 84/130) and 35.4% (*N* = 46/130) in the delayed period. No cases had peak LFT elevations in the late period. Aspartate transaminase elevation was identified at rates similar to ALT elevation, although the temporal distribution of peak AST elevation appeared to favor a slightly earlier onset (Figure 2). Transaminase elevation appeared to resolve largely within the follow-up period (Supplemental Figure 1).

The median (IQR) ALT/AST ratio of all LFT measurements on Day 0 was 1.0 (0.7 to 1.2). The ALT/AST ratio progressively increased to consistently > 1 after Day 4. There was no difference in the ALT/AST ratio based on infective species.

Elevated bilirubin, ALT, and AST levels were observed significantly more frequently in *P. falciparum* than in *P. vivax* cases (Table 2).

Of all cases with LFTs undertaken, there were 54 cases with normal (*N* = 11/861) or mildly elevated (*N* = 43/861) LFTs on the day of diagnosis that subsequently went on to develop moderate or greater abnormalities. Liver function test results by day of collection for these cases are presented in Supplemental Figure 1.

### Comparison of cases with serial normal transaminases, early and delayed transaminase elevations.

A total of 173 cases were compared to identify factors potentially contributing to the presence and timing of transaminase elevation. Early transaminase elevation was identified in 84/173 cases, delayed elevation in 46/173 cases, and normal measurements in 43/173 cases (Table 3). No late transaminase elevations were identified. All LFT results by day of sample collection are presented for each group in Supplemental Figure 2.

**Table 3:** Comparison of demographic factors, clinical factors, and baseline liver function tests in serial normal transaminases, early and delayed transaminase elevation groups

	Serial normal transaminases (*N* = 43)	Early transaminase elevation (*N* = 84)	Delayed transaminase elevation (*N* = 46)	*P* value
Age (years) median, IQR (*N*)	40.2, 25.9–51.1 (43)	37.4, 25.0–49.2 (84)	35.5, 26.8–47.8 (46)	0.909*
Gender (male) % (*n*/*N*)	74.4 (32/43)	70.2 (59/84)	71.7 (33/46)	0.885†
Cases infected with *Plasmodium falciparum* % (*n*/*N*)	58.1 (25/43)	57.1 (48/84)	76.1 (35/46)	0.082†
Parasitemia (parasites/µL) median, IQR (*N*)	12,000, 3,775–110,000 (38)	5,900, 1,375–44,000 (74)	12,500, 1,650–85,750 (42)	–
Median log parasitemia	4.08	3.77	4.10	0.483‡
Region % (*n*/*N*)				**< 0.001**†
South Asia/Oceania	37.2 (16/43)	31.0 (26/84)	13.0 (6/46)	
Subcontinent	11.6 (5/43)	1.2 (1/84)	2.2 (1/46)	
Africa	41.9 (18/43)	8.3 (7/84)	15.2 (7/46)	
Multiple regions	7.0 (3/43)	1.2 (1/84)	0 (0/46)	
Unknown/not recorded	2.3 (1/43)	58.3 (49/84)	69.6 (32/46)	
Treatment (artemesinin containing) % (*n*/*N*)	85.7 (36/42)	88.6 (31/35)	100 (14/14)	0.333†
Treatment delay (days) median, IQR (*N*)	1, 0–2 (41)	1, 0–1 (34)	1, 0–2 (15)	0.769*
Other infection (any testing) % (*n*/*N*)				**0.005**†
Positive	23.2 (10/43)	13.1 (11/84)	4.3 (2/46)	
Negative	25.6 (11/43)	38.1 (32/84)	60.9 (28/46)	
Unknown/not tested	51.2 (22/43)	48.8 (41/84)	34.8 (16/46)	
Bilirubin at diagnosis (×ULN) median, IQR (*N*)	1.5, 1.0–2.3 (42)	1.4, 0.9–2.0 (79)	1.5, 0.9–2.7 (42)	–
Median log bilirubin at diagnosis	0.18	0.15	0.18	0.710*
ALT at diagnosis (×ULN) median, IQR (*N*)	0.8, 0.6–1.2 (42)	3.5, 2.9–4.4 (80)	1.7, 1.1–3.0 (42)	–
Median log ALT at diagnosis	−0.09	0.54	0.24	**< 0.001**‡
AST at diagnosis (×ULN) median, IQR (*N*)	1.0, 0.8–1.3 (41)	3.0, 2.3–4.2 (78)	2.2, 1.5–3.6 (40)	–
Median log AST at diagnosis	0	0.50	0.34	**< 0.001**‡
ALT/AST at diagnosis (ratio) median, IQR (*N*)	1.0, 0.7–1.2 (41)	1.2, 0.9–1.6 (78)	1.1, 0.8–1.3 (40)	0.003*

ALT = alanine transaminase; AST = aspartate transaminase; IQR = interquartile range; ULN = upper limit of normal. *N* refers to the total sample size, and *n* refers to the number of cases with data available on the particular demographic or clinical factor. Alanine transaminase/aspartate transaminase was calculated using unadjusted data for each patient at diagnosis. Values reaching statistical significance were formatted in bold.

* Kruskal–Wallis test.

† Chi-squared test.

‡ One-way analysis of variance.

Transaminase results at diagnosis differed between all groups, highest in the early transaminase elevation group (log ALT × ULN, *P* < 0.001 and log AST × ULN, *P* < 0.001). Furthermore, the ALT/AST ratio at diagnosis was also higher in the early transaminase elevation group (*P* = 0.003). Region of malaria acquisition (*P* < 0.001) and positive testing for another infection potentially contributing to LFT abnormalities (*P* = 0.005) were the only nontransaminase indices that differed between groups. Data on region of acquisition was more readily available in the normal transaminase group, which had fewer “unknown” category results than the other groups. Positive testing for other infections was also most common in the normal transaminase group. All groups demonstrated a similar age and gender distribution. Furthermore, *P. falciparum* was the most common infective species across all groups, and parasitemia at presentation was similar. The median delay to treatment was 1 day after diagnosis, and the majority of cases received an artemisinin-containing regimen (Table 3).

Multivariate analysis by paired comparison of normal transaminases and early transaminase elevation unsurprisingly found log ALT on the day of diagnosis had an extremely strong association with early transaminase elevation (LR = 84, *P* < 0.001). Region of acquisition (LR = 27, *P* < 0.001), lower log bilirubin level on the day of diagnosis (LR = 15, *P* < 0.001), higher rates of *P. vivax* infection (LR = 12, *P* < 0.001), and female gender (LR = 8, *P* = 0.004) were also associated with early transaminase elevation compared with the normal transaminases group. Delayed transaminase elevation was associated with a higher log ALT level on the day of diagnosis compared with normal transaminases (LR = 17, *P* < 0.001). Region of acquisition was also different between these groups (LR = 25, *P* < 0.001). Comparison of the delayed and early transaminase groups found that higher log ALT on the day of diagnosis (LR = 21, *P* < 0.001) and positive testing for other infections (LR = 11, *P* = 0.004) were associated with early transaminase elevation.

## DISCUSSION

In this audit, we have identified rates of elevated bilirubin and malarial hepatopathy consistent with other reports.^1–3,8,9^ These abnormalities are transient, more frequently observed in *P. falciparum* infection than in other *Plasmodium* species, and may vary widely in severity (up to > 25 × ULN). We have also identified a relatively large proportion of cases with elevated transaminases after diagnosis and treatment that has not been well described previously in the naturally acquired malaria literature. Although elevations in bilirubin levels appear to peak and resolve soon after treatment, peak transaminase elevations may not occur simultaneously. This may represent malaria-associated liver effects after hemolysis, which could explain the progressive increase in the ALT/AST ratio observed with time for all cases, as the hemolysis-related AST level decreases while the hepatocyte-specific ALT level increases.

Analysis of factors potentially contributing to the presence and timing of transaminase elevation predictably demonstrated that the ALT level on the day of diagnosis had the greatest association with the timing of transaminase elevation. This is unsurprising for cases with LFTs peaking in the first 3 days and is an inherent limitation of using this variable. Interestingly, those who experienced delayed elevation had a higher ALT level on the day of diagnosis compared with the normal transaminases group, suggesting that hepatic injury was already underway. Given that treatment was instituted soon after diagnosis, this delayed transaminase elevation may suggest continued hepatocyte injury in the postparasite clearance period. Serum ALT typically responds to hepatocyte injury within hours after insult rather than days.^35^ In this study, delayed ALT elevation peaking 4–11 days after diagnosis was common. This could represent an underdescribed burden of malaria-associated liver injury, especially given that the frequency of LFT measurements is reduced with time since diagnosis, and LFTs are frequently not performed serially if normal or only mildly elevated at diagnosis.

Treatment regimen and the timing of institution of treatment after diagnosis were not associated with the timing of peak transaminase elevation. This is in contrast to other studies associating artemisinin-containing treatment regimens, and presumably more rapid parasite clearance, with LFT elevations.^28^ *Plasmodium falciparum* infection was associated with a higher frequency of abnormal LFTs overall. However, when the three time period groups were compared, the proportion of *P. falciparum* cases compared with *P. vivax* cases was only different between the normal and early transaminase elevation groups. Interestingly, infection with another organism that may cause liver injury such as dengue or viral hepatitis was more common in the early transaminase elevation group compared with the delayed transaminase group, which may have contributed to earlier peak hepatocyte injury. Based on the available data, the normal transaminases group had the highest absolute rate of coinfection, which is not biologically plausible. Differences between groups in region of malaria acquisition are similarly difficult to interpret, given the low number of cases, high number of categories, and discrepancies in data availability between comparison groups but could be related to either population or parasite strain-related differences.

Although this analysis was limited, there do not seem to be clear demographic or clinical differences between groups that are predictive of liver injury. We hypothesize that other individual-specific factors may confer susceptibility to hepatocyte injury. This may include host ability to manage reactive oxygen species generated by heme breakdown after erythrocyte recycling in the liver and the degree of proinflammatory immune response to infection. In this population, the bilirubin level at the time of diagnosis was only different between normal and early transaminase elevation groups, which may support the hypothesis that the burden of hemolysis alone does not modulate the timing of transaminase elevation. This is supported by mechanistic studies of malaria-associated liver injury in mouse models, which demonstrate that heme-mediated oxidative damage is promoted by tumor necrosis factor (TNF) in a synergistic fashion.^36–40^

It is clear that the pattern of abnormal LFTs is variable in malaria infection, ranging from acute hepatopathy associated with jaundice and poor prognosis, to asymptomatic transaminase elevation. Differentiating malaria-associated liver injury from drug-induced liver injury is important both at the bedside and in the context of antimalarial drug development. This study has found that elevations in bilirubin levels and malarial hepatopathy occur exclusively in the few days after diagnosis and treatment, which may be a useful feature to help differentiate malaria associated changes from potentially more delayed significant drug-induced liver injury meeting Hy’s Law. The identification of delayed transaminase elevation in this study provides more of a challenge, given that this likely forms part of the normal pathophysiology of disease but varies between individuals. This adds an extra layer of complexity to interpreting safety data from clinical trials undertaken for antimalarial drug development. For example, in field studies of pyronaridine–artesunate in uncomplicated malaria, mild transient transaminase elevations were reported on Days 3 and 7 after treatment, which were attributed to direct low-level drug toxicity.^41^ These elevations occurred in the same time period as the delayed transaminase elevations described in this study, which followed a range of antimalarial treatments, and are of a similar severity. If delayed transaminase elevation is truly part of the spectrum of malaria-associated liver injury as we propose, it is clear that assigning causality in the trial setting will continue to be challenging. This problem will exist at least until either individual predisposing factors for malaria-associated liver injury are better understood or biomarkers to distinguish between drug-induced liver injury and malarial hepatopathy are identified.

By including all cases of falciparum and vivax malaria irrespective of initial LFT results and hospital or community setting, this study population is broader than that typically reported. Nonetheless, the retrospective nature of this audit means that the datasets are incomplete, which is a major limitation of this report. Furthermore, although it is assumed that the study population is largely malaria naive, given that Australia is malaria-free, this could not be confirmed from the available data. The use of potentially hepatotoxic medications such as acetaminophen often used in malaria patients, and which may affect LFT results, was not recorded in this study. Many confirmed cases of malaria did not have LFTs for review. In those who had LFTs collected, there is likely a selection bias toward sampling in more severe syndromes and serial testing in those with already abnormal results. This may explain why those with any LFTs collected were older and had a higher proportion of falciparum malaria compared with those with no LFTs. Although this may result in overestimation of the rate of abnormal LFTs in early malarial illness, it may also result in underestimation of delayed transaminase elevations. The absolute rate of delayed transaminase elevation in malaria cannot be determined from this analysis, as the delayed time period was used to find cases. A prospective study with LFT sampling after completion of treatment is required to measure the incidence of this phenomenon. Genotyping heme oxygenase 1—the enzyme that catalyzes heme degradation—in patients, and measuring circulating TNF levels at diagnosis and after treatment, could be used to test the hypothesis that individual heme metabolism and TNF-associated inflammatory response may confer susceptibility to malaria-associated liver injury.

## CONCLUSION

Liver function test abnormalities occur commonly in persons infected with various plasmodia and are likely an inherent although variable aspect of human malaria. Elevated transaminase levels due to malaria are transient, may be delayed beyond antimalarial treatment, and may not occur simultaneously with bilirubin changes. No clinical or demographic factors are clearly related to transaminase elevation; however, extrapolation of mouse-model studies may suggest that individual variation in inflammatory response and the efficiency of heme breakdown could play a role in transaminase elevation.

## Supplementary Material

Supplemental Figures and Tables

## References

[1] Jain A Kaushik R Kaushik RM, 2016 Malarial hepatopathy: clinical profile and association with other malarial complications. Acta Trop 159: 95–105. 27019056 10.1016/j.actatropica.2016.03.031

[2] Anand AC Ramji C Narula AS Singh W, 1992 Malarial hepatitis: a heterogeneous syndrome? Natl Med J India 5: 59–62. 1304265

[3] Mehta SR Naidu G Chandar V Singh IP Johri S Ahuja RC, 1989 Falciparum malaria—present day problems. An experience with 425 cases. J Assoc Physicians India 37: 264–267. 2693436

[4] Murthy GL Sahay RK Sreenivas DV Sundaram C Shantaram V, 1998 Hepatitis in falciparum malaria. Trop Gastroenterol 19: 152–154. 10228440

[5] Mazumder R Mishra RK Mazumder H Mukherjee P, 2002 Jaundice in falciparum malaria—some prospective observations. J Indian Med Assoc 100: 312–314. 12418632

[6] Wilairatana P Looareesuwan S Charoenlarp P, 1994 Liver profile changes and complications in jaundiced patients with falciparum malaria. Trop Med Parasitol 45: 298–302. 7716391

[7] Ramachandran S Perera MV, 1976 Jaundice and hepatomegaly in primary malaria. J Trop Med Hyg 79: 207–210. 794514

[8] Hazra BR Chowdhury RS Saha SK Ghosh MB Mazumder AK, 1998 Changing scenario of malaria: a study at Calcutta. Indian J Malariol 35: 111–116. 10448229

[9] Tangpukdee N Thanachartwet V Krudsood S Luplertlop N Pornpininworakij K Chalermrut K Phokham S Kano S Looareesuwan S Wilairatana P, 2006 Minor liver profile dysfunctions in *Plasmodium vivax*, *P. malariae* and *P. ovale* patients and normalization after treatment. Korean J Parasitol 44: 295–302. 17170571 10.3347/kjp.2006.44.4.295 PMC2559128

[10] Mishra SK Mohanty S Das BS Patnaik JK Satpathy SK Mohanty D Bose TK, 1992 Hepatic changes in *P. falciparum* malaria. Indian J Malariol 29: 167–171. 1286732

[11] Gupta UC Kataria ML, 1993 *Plasmodium falciparum* hepatitis during malaria epidemic. J Assoc Physicians India 41: 292. 7695667

[12] Kochar DK Agarwal P Kochar SK Jain R Rawat N Pokharna RK Kachhawa S Srivastava T, 2003 Hepatocyte dysfunction and hepatic encephalopathy in *Plasmodium falciparum* malaria. QJM 96: 505–512. 12881593 10.1093/qjmed/hcg091

[13] Abro AH Ustadi AM Abro HA Abdou AS Younis NJ Akaila SI, 2009 Jaundice with hepatic dysfunction in *P. falciparum* malaria. J Coll Physicians Surg Pak 19: 363–366. 19486575

[14] Patwari A Aneja S Berry A Ghosh S, 1979 Hepatic dysfunction in childhood malaria. Arch Dis Child 54: 139–141. 373643 10.1136/adc.54.2.139 PMC1545372

[15] Anand AC Puri P, 2005 Jaundice in malaria. J Gastroenterol Hepatol 20: 1322–1332. 16105116 10.1111/j.1440-1746.2005.03884.x

[16] Kochar DK Kaswan K Kochar SK Sirohi P Pal M Kochar A Agrawal RP Das A, 2006 A comparative study of regression of jaundice in patients of malaria and acute viral hepatitis. J Vector Borne Dis 43: 123–129. 17024861

[17] Fazil A Vernekar PV Geriani D Pant S Senthilkumaran S Anwar N Prabhu A Menezes RG, 2013 Clinical profile and complication of malaria hepatopathy. J Infect Public Health 6: 383–388. 23999343 10.1016/j.jiph.2013.04.003

[18] Saya RP Debabrata G Saya GK, 2012 Malarial hepatopathy and its outcome in India. N Am J Med Sci 4: 449. 23112964 10.4103/1947-2714.101981 PMC3482774

[19] Bhalla A Suri V Singh V, 2006 Malarial hepatopathy. J Postgrad Med 52: 315–320. 17102560

[20] Temple R, 2006 Hy’s law: predicting serious hepatotoxicity. Pharmacoepidemiol Drug Saf 15: 241–243. 16552790 10.1002/pds.1211

[21] Bag S Samal GC Deep N Patra UC Nayak M Meher LK, 1994 Complicated falciparum malaria. Indian Pediatr 31: 821–825. 7890345

[22] Shah S Ali L Sattar RA Aziz T Ansari T Ara J, 2009 Malarial hepatopathy in falciparum malaria. J Coll Physicians Surg Pak 19: 367–370. 19486576

[23] Kachawaha S Pokharana R Rawat N Garg P Badjatiya H Kochar DK, 2003 Ultrasonography in malarial hepatitis. Indian J Gastroenterol 22: 110. 12839391

[24] Chawla LS Sidhu G Sabharwal BD Bhatia KL Sood A, 1989 Jaundice in *Plasmodium falciparum*. J Assoc Physicians India 37: 390–391. 2687227

[25] Joshi YK Tandon BN Acharya SK Babu S Tandon M, 1986 Acute hepatic failure due to *Plasmodium falciparum* liver injury. Liver 6: 357–360. 3553821 10.1111/j.1600-0676.1986.tb00304.x

[26] McMahon AE Kelsey JE Derauf DE, 1954 Hepatitis of malarial origin: clinical and pathologic study of fifty-four Korean veterans. AMA Arch Intern Med 93: 379–386. 13123562 10.1001/archinte.1954.00240270065006

[27] Kleeberg J Birnbaum D, 1948 Studies on liver damage in acute malaria. Trans R Soc Trop Med Hyg 41: 555–566. 18902145 10.1016/s0035-9203(48)90831-1

[28] Silva-Pinto A Ruas R Almeida F Duro R Silva A Abreu C Sarmento A, 2017 Artemether–lumefantrine and liver enzyme abnormalities in non-severe *Plasmodium falciparum* malaria in returned travellers: a retrospective comparative study with quinine-doxycycline in a Portuguese centre. Malar J 16: 43. 28122572 10.1186/s12936-017-1698-y PMC5264472

[29] McCarthy JS 2016 A phase II pilot trial to evaluate safety and efficacy of ferroquine against early *Plasmodium falciparum* in an induced blood-stage malaria infection study. Malar J 15: 469. 27624471 10.1186/s12936-016-1511-3 PMC5022189

[30] Griffin P 2016 Safety and reproducibility of a clinical trial system using induced blood stage *Plasmodium vivax* infection and its potential as a model to evaluate malaria transmission. PLoS Negl Trop Dis 10: e0005139. 27930652 10.1371/journal.pntd.0005139 PMC5145139

[31] Nyunt MM Hendrix CW Bakshi RP Kumar N Shapiro TA, 2009 Phase I/II evaluation of the prophylactic antimalarial activity of pafuramidine in healthy volunteers challenged with *Plasmodium falciparum* sporozoites. Am J Trop Med Hyg 80: 528–535. 19346370 PMC2763313

[32] Epstein JE Rao S Williams F Freilich D Luke T Sedegah M de la Vega P Sacci J Richie TL Hoffman SL, 2007 Safety and clinical outcome of experimental challenge of human volunteers with *Plasmodium falciparum*-infected mosquitoes: an update. J Infect Dis 196: 145–154. 17538895 10.1086/518510

[33] Watkins PB Kaplowitz N Slattery JT Colonese CR Colucci SV Stewart PW Harris SC, 2006 Aminotransferase elevations in healthy adults receiving 4 grams of acetaminophen daily: a randomized controlled trial. JAMA 296: 87–93. 16820551 10.1001/jama.296.1.87

[34] WHO Offset Publication, 1979 *WHO Handbook for Reporting Results of Cancer Treatment.* Geneva, Switzerland: World Health Organization, 48, 16–21.

[35] Antoine DJ 2013 Mechanistic biomarkers provide early and sensitive detection of acetaminophen-induced acute liver injury at first presentation to hospital. Hepatology 58: 777–787. 23390034 10.1002/hep.26294 PMC3842113

[36] Seixas E Oliveira P Moura Nunes JF Coutinho A, 2008 An experimental model for fatal malaria due to TNF-alpha-dependent hepatic damage. Parasitology 135: 683–690. 18377697 10.1017/S0031182008004344

[37] Seixas E 2009 Heme oxygenase-1 affords protection against noncerebral forms of severe malaria. Proc Natl Acad Sci USA 106: 15837–15842. 19706490 10.1073/pnas.0903419106 PMC2728109

[38] Oliveira-Lima OC Bernardes D Xavier Pinto MC Esteves Arantes RM Carvalho-Tavares J, 2013 Mice lacking inducible nitric oxide synthase develop exacerbated hepatic inflammatory responses induced by *Plasmodium berghei* NK65 infection. Microbes Infect 15: 903–910. 23988520 10.1016/j.micinf.2013.08.001

[39] Dey S Mazumder S Siddiqui AA Iqbal MS Banerjee C Sarkar S De R Goyal M Bindu S Bandyopadhyay U, 2014 Association of heme oxygenase 1 with the restoration of liver function after damage in murine malaria by *Plasmodium yoelii*. Infect Immun 82: 3113–3126. 24818663 10.1128/IAI.01598-14 PMC4136231

[40] Guha M Kumar S Choubey V Maity P Bandyopadhyay U, 2006 Apoptosis in liver during malaria: role of oxidative stress and implication of mitochondrial pathway. FASEB J 20: 1224–1226. 16603602 10.1096/fj.05-5338fje

[41] Duparc S Borghini-Fuhrer I Craft CJ Arbe-Barnes S Miller RM Shin CS Fleckenstein L, 2013 Safety and efficacy of pyronaridine-artesunate in uncomplicated acute malaria: an integrated analysis of individual patient data from six randomized clinical trials. Malar J 12: 70. 23433102 10.1186/1475-2875-12-70 PMC3598551

