# Supplementary material for: The Dynamics of Liver Function Test Abnormalities after Malaria Infection: A Retrospective Observational Study
Source: Am J Trop Med Hyg. 2018 Feb 12;98(4):1113–9. doi: 10.4269/ajtmh.17-0754 (PMC5928828; doi:10.4269/ajtmh.17-0754)
Supplement: Supplementary file 1 [file tpmd170754.SD1.pdf]

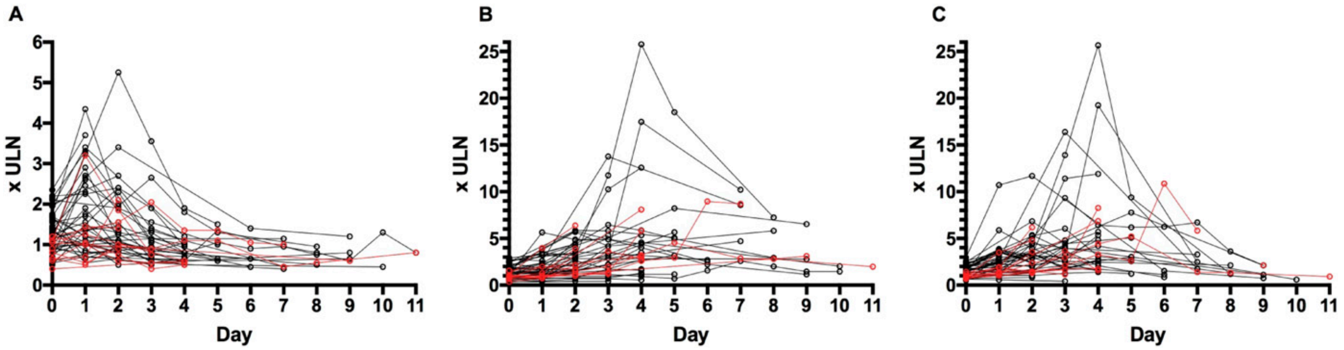

Supplemental Figure 1. Liver function test (LFT) results by day of sample collection for all cases with normal LFTs (red, N = 11/861) or mild elevations only (black, N = 43/861) on the day of diagnosis that went on to have moderate or greater abnormalities in LFTs in the follow-up period. (**A**) Bilirubin. (**B**) ALT. (**C**) AST. Bilirubin grading: normal < 1.25 × ULN and mild 1.25–2.5 × ULN. ALT and AST grading: normal < 1.25 × ULN and mild 1.25–3 × ULN. Day 0 was the day of diagnosis. ALT = alanine transaminase; AST = aspartate transaminase; ULN = upper limit of normal.

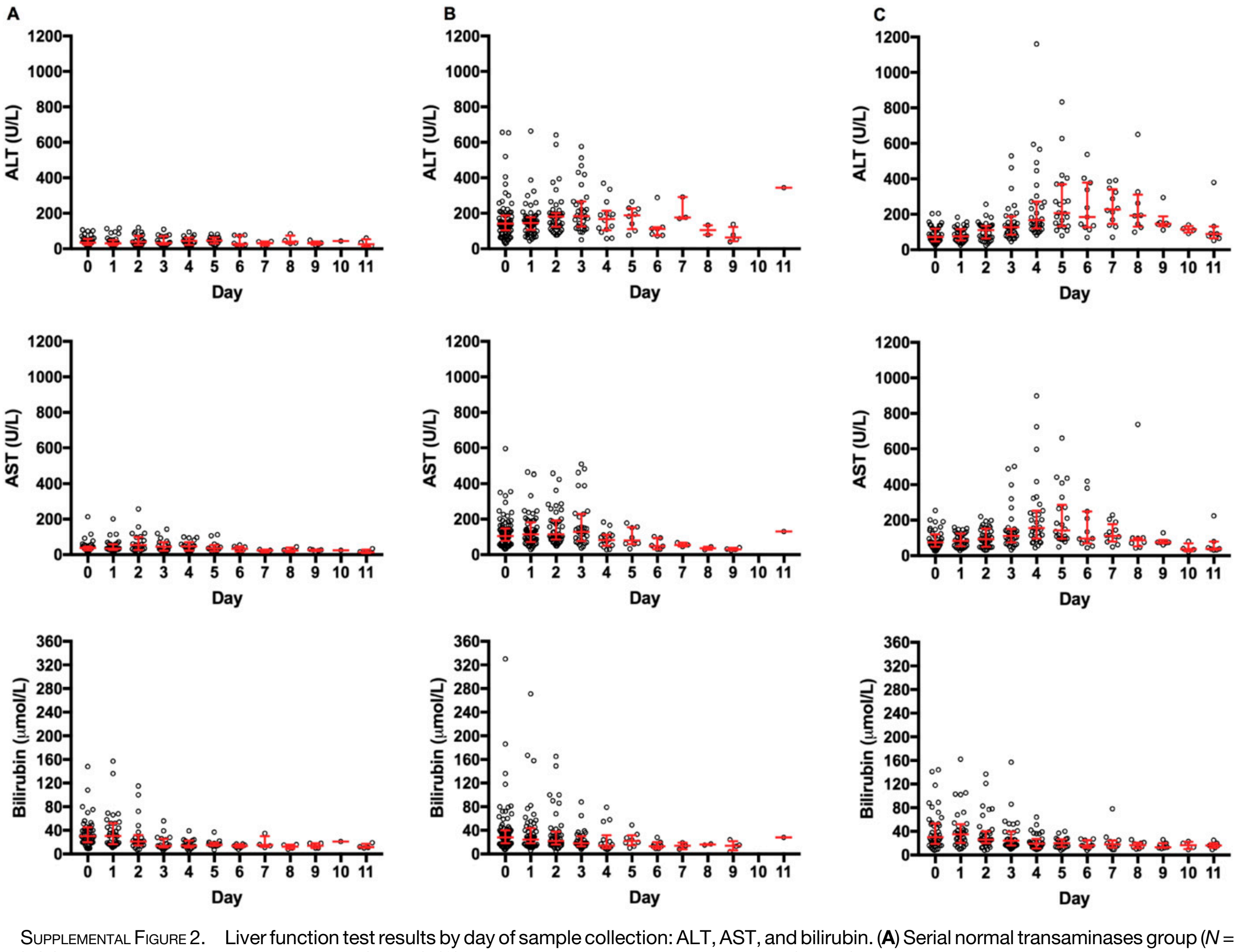

Supplemental Figure 2. Liver function test results by day of sample collection: ALT, AST, and bilirubin. (**A**) Serial normal transaminases group (N = 43). (**B**) Early transaminase elevation group (N = 84). (**C**) Delayed transaminase elevation group (N = 46). Red bars represent median and interquartile ranges of measurements for each day. ALT = alanine transaminase; AST = aspartate transaminase.

Supplemental Table 1

Age and gender reference ranges for ALT, AST, and total bilirubin

Age	Gender	Normal range
ALT		
0–30 days	M	< 25
30–365 days	M	< 35
1–3 years	M	5–30
3–6 years	M	5–20
6–9 years	M	5–25
9–18 years	M	5–30
18–120 years	M	< 45
0–30 days	F	< 25
30–365 days	F	< 30
1–3 years	F	5–30
3–6 years	F	5–25
6–9 years	F	5–25
9–18 years	F	5–20
18–120 years	F	< 34
AST		
0–30 days	M	< 51
30–365 days	M	< 65
1–3 years	M	< 56
3–6 years	M	< 48
6–9 years	M	< 42
9–12 years	M	< 38
12–15 years	M	< 39
15–18 years	M	< 39
0–30 days	F	< 49
30–365 days	F	< 79
1–3 years	F	< 69
3–6 years	F	< 59
6–9 years	F	< 41
9–12 years	F	< 37
12–15 years	F	< 32
15–18 years	F	< 30
18–120 years	M	< 35
18–120 years	F	< 31
Total bilirubin		
0–1 days	M + F	< 100
1–2 days	M + F	< 140
3–5 days	M + F	< 200
5–11 days	M + F	< 100
11 days to 120 years	M + F	< 20

ALT = alanine transaminase; AST = aspartate transaminase; F = female; M = male. ALT and AST are measured in Units/L. Total bilirubin is measured in μM/L.
